# Significance of gene mutations in the Wnt signaling pathway in traditional serrated adenomas of the colon and rectum

**DOI:** 10.1371/journal.pone.0229262

**Published:** 2020-02-24

**Authors:** Hiroyoshi Nakanishi, Takeshi Sawada, Yasuharu Kaizaki, Ryosuke Ota, Hiromu Suzuki, Eiichiro Yamamoto, Hironori Aoki, Makoto Eizuka, Kenkei Hasatani, Naoki Takahashi, Satoko Inagaki, Masahide Ebi, Hiroyuki Kato, Eiji Kubota, Hiromi Kataoka, Satoru Takahashi, Takashi Tokino, Toshinari Minamoto, Tamotsu Sugai, Yasushi Sasaki

**Affiliations:** 1 Division of Translational and Clinical Oncology, Cancer Research Institute, Kanazawa University, Kanazawa, Japan; 2 Department of Gastroenterology and Metabolism, Nagoya City University Graduate School of Medical Sciences, Nagoya, Japan; 3 Department of Pathology, Fukui Prefectural Hospital, Fukui, Japan; 4 Department of Molecular Biology, Sapporo Medical University School of Medicine, Sapporo, Japan; 5 Department of Molecular Diagnostic Pathology, Iwate Medical University School of Medicine, Morioka, Japan; 6 Department of Gastroenterology, Fukui Prefectural Hospital, Fukui, Japan; 7 Department of Gastroenterology, Saitama Cancer Center, Saitama, Japan; 8 Department of Advanced Research in Community Medicine, Kanazawa University Graduate School of Medical Sciences, Kanazawa, Japan; 9 Department of Gastroenterology, Aichi Medical University, Nagakute, Japan; 10 Department of Experimental Pathology and Tumor Biology, Nagoya City University Graduate School of Medical Sciences, Nagoya, Japan; 11 Department of Medical Genome Sciences, Research Institute for Frontier Medicine, Sapporo Medical University, Sapporo, Japan; 12 Division of Biology, Department of Liberal Arts and Sciences, Center for Medical Education, Sapporo Medical University, Sapporo, Japan; University of Kentucky, UNITED STATES

## Abstract

Recent studies have shown that colorectal serrated lesions, which include sessile serrated adenomas (SSAs) and traditional serrated adenomas (TSAs), are precursors of colorectal cancer. However, the molecular mechanisms underlying the carcinogenesis, particularly in TSAs, remain largely uncharacterized. To clarify their molecular and clinicopathological characteristics, we performed mutation and methylation analyses of cancer-associated genes in 78 serrated lesions, including TSAs, SSAs and microvesicular hyperplastic polyps. Target exon sequence analysis was performed with 39 genes, including genes known to be frequently mutated in colorectal cancers and/or serrated lesions. We also used bisulfite pyrosequencing to assess the methylation status of various cancer-associated genes and marker genes of the CpG island methylator phenotype (CIMP). The prevalence of mutations in genes associated with Wnt signaling was significantly higher in TSAs than SSAs (65% vs. 28%, p < 0.01). Among those, *RNF43* mutations were observed in 38% of TSAs and 17% of SSAs. In immunohistochemical studies of 39 serrated lesions, the prevalence of abnormal nuclear β-catenin accumulation was significantly higher in TSAs (57%) than SSAs (8%) (P = 0.01). *SMOC1* methylation was detected in 54% of TSAs but in no SSAs (p < 0.01). Additionally, *SMOC1* methylation was more prevalent among TSAs with *KRAS* mutation (82%) than with *BRAF* mutation (38%, p = 0.03). Lesions with CIMP-high or *RNF43* mutations were detected only in TSAs with *BRAF* mutation, suggesting two distinct carcinogenic pathways in TSAs. Mutations in genes associated with Wnt signaling play a greater role in the carcinogenesis of TSAs than SSAs.

## Introduction

Colorectal cancer (CRC) is a major cause of cancer-related death worldwide. Recent molecular pathological studies, including The Cancer Genome Atlas project, have shown that CRCs are heterogeneous diseases that arise via different molecular pathways [1,2]. Most (80–85%) sporadic CRCs are classified as non-hypermutated or microsatellite stable (MSS) tumors and develop through accumulation of multiple genetic and epigenetic alterations [1,2], including mutation of oncogenes and tumor suppressor genes, as well as chromosomal instability [3,4]. The remaining 15–20% of sporadic CRCs are classified as hypermutated tumors and mainly exhibit microsatellite instability (MSI) and concurrent hypermethylation in multiple loci [2], which is referred to as the CpG island methylator phenotype (CIMP) and is closely associated with *BRAF* mutation [5–7].

Recent studies of the molecular and clinicopathological characteristics of colorectal premalignant lesions have provided insight into the pathogenesis of CRCs as well as clues to prevention and treatment [8–10]. Since establishing the pathological classification of serrated colorectal lesions as hyperplastic polyps, traditional serrated adenomas (TSAs) or sessile serrated adenomas (SSAs) [11–13], a number of studies have demonstrated that SSAs are associated with *BRAF* mutation and CIMP, and that they are precursors of MSI-positive CRCs, which are frequently located in the proximal colon [6,9,10,14–17]. However, less is known about the biological and clinical characteristics of TSAs, although they are also considered to be premalignant lesions and reportedly exhibit *BRAF* or *KRAS* mutations and aberrant DNA methylation [16–21]. In addition, several recent studies have reported *PTPRK-RSPO3* fusion and somatic mutations of *RNF43* in TSAs [22–24]. Because only a small number of analyses have investigated gene mutations in serrated lesions [23,25,26], the molecular mechanisms underlying carcinogenesis, especially in TSAs, are still not well characterized. Although it has been suggested based on immunohistochemical studies that dysregulation of the Wnt signaling pathway contributes to carcinogenesis in serrated lesions [20,21,23,27–29], mutation of individual gene within this pathway have not been investigated.

To clarify the molecular and clinicopathological characteristics of colorectal serrated lesions, we assessed the mutation of genes associated with Wnt signaling as well as other genes reportedly mutated in serrated lesions [26] and advanced CRCs [2]. We also investigated mutations in genes associated with oncogene-induced senescence, which have been reported as germline mutations in patients with multiple SSAs [30], and we performed immunohistochemical studies of β-catenin expression to assess activation of the Wnt signaling pathway. Finally, we investigated DNA methylation of cancer-associated genes including *SMOC1* as well as CIMP marker genes in TSAs because it was reported that *SMOC1* is specifically methylated in TSAs [31].

## Materials and methods

### Patients and tissue samples

Specimens of colorectal serrated lesions (n = 78) were obtained from 78 Japanese patients who underwent endoscopic mucosal resection at Nagoya City University Hospital, Fukui Prefectural Hospital, or Komatsu Municipal Hospital. This study was approved by the Institutional Review Board at each hospital as well as Kanazawa University and Sapporo Medical University.

### Endoscopic analysis

High-resolution magnifying endoscopes (CF260AZI; Olympus, Tokyo, Japan) were used for all colonoscopic examinations. The morphology of colorectal lesions was determined according to the Paris classification [32]. All lesions detected during colonoscopy were observed at high magnification using indigo carmine dye, after which samples were collected through endoscopic mucosal resection for histological analysis. Tumor locations were defined as proximal colon (cecum, ascending colon, transverse colon) or distal colon (descending colon, sigmoid colon, and rectum).

### Histological analysis

Histological diagnosis of tumors was done at each facility, after which the histological findings for all specimens were reviewed by a board certified pathologist (Sugai T) who was blinded to the clinical and molecular information. Serrated lesions, including microvesicular hyperplastic polyps (MVHPs), SSAs and TSAs, were classified according to WHO classification criteria [33]. Mixed serrated lesion composed of TSA and tubulovillous adenoma was classified as TSA in our analyses. The clinicopathological features of the lesions are summarized in Table 1.

**Table 1:** Clinicopathological features of the serrated lesions in this study.

**Patients (n = 78)**	
**Age (y, mean ± SD)**	65.3 ± 10.9
**Sex, n (%)**	
Male	51 (65)
Female	27 (35)
**Lesions (n = 78)**	
**Location, n (%)**	
Proximal	42 (54)
Distal	36 (46)
**Bowel subsites, n (%)**	
Cecum	13 (17)
Ascending colon	22 (28)
Transverse colon	7 (9)
Descending colon	5 (6)
Sigmoid colon	20 (26)
Rectum	11 (14)
**Morphology, n (%)**	
0-Ip	15 (19)
0-Is	47 (60)
0-IIa	16 (21)
**Histology, n (%)**	
MVHP	23 (30)
TSA	36 (46)
TSA + TVA	1 (1)
SSA	18 (23)

MVHP, microvesicular hyperplastic polyp; TSA, traditional serrated adenoma; TVA, tubulovillous adenoma; SSA, sessile serrated adenoma.

### DNA preparation

DNA was isolated from formalin-fixed, paraffin-embedded (FFPE) tissue sections using a QIAamp DNA FFPE Tissue kit (Qiagen, Hilden, Germany) following the manufacturer’s instructions. A TaqMan RNase P Detection Reagents kit (Thermo Fisher Scientific, Waltham, MA) was used to quantify the purified DNA.

### Semiconductor-based next-generation sequencing

A customized panel, encompassing all the exons of 39 cancer-related genes, including genes frequently reported to be involved in advanced CRCs and serrated lesions [2,25,26], was created using the Ion Torrent System with an Ion AmpliSeq Designer (Thermo Fisher Scientific) (S1 Fig). Genes within oncogene-induced senescence pathways detected in patients with multiple serrated polyps were also investigated [30]. The assay design consisted of 1,455 amplicons ranging from 125 to 175 bp in length, covering 94% of the 112.8 kb target sequence.

Library preparation and sequencing with the Ion Torrent sequencer were performed as previously described [34–36]. The templates were sequenced after emulsion PCR was performed with 24 samples per Ion PI chip using the Ion PI HI-Q Chef kit (Thermo Fisher Scientific).

### Identification of somatic mutations and copy number variations (CNVs)

Human genome build 19 (hg19) was used as a reference. Signal processing, mapping to the hg19 reference, and quality control were performed using Torrent Suite version 5.0 (Thermo Fisher Scientific). Somatic mutations (point mutations, insertions, and deletions) were detected using Ion Reporter Software 5.0 (Thermo Fisher Scientific). Because matched normal controls were not available, the control sequence data provided by Thermo Fisher Scientific were used as a control. Pathogenic status of the variant was stated if it was a missense variant with < 0.1% global minor allele frequency in dbSNP or the 1000 Genomes Project database and/or the variant was registered as pathogenic in ClinVar or COSMIC databases. Variants with allele frequencies between 0.4 and 0.6 or > 0.9 were considered germline variants unless listed as a pathogenic variant. Additionally, if the same variants were detected in multiple samples, these variants were considered germline variants unless occurring at a known hotspot variant in databases. Integrative Genomics Viewer (IGV) software (http://software.broadinstitute.org/software/igv/) was used to filter out possible strand-specific errors, such as a mutation that was detected in the forward or reverse DNA strand but not in both strands. CNV detection was also performed with the Ion Reporter Software using an algorithm based on the Hidden Markov Model. Recurrent genomic regions with CNVs were identified using copy numbers greater than 3 and less than 1 for gains and losses, respectively.

### DNA methylation analysis

DNA methylation was analyzed using bisulfite pyrosequencing as described previously [37,38]. Briefly, genomic DNA (1 μg) was modified with sodium bisulfite using an EpiTect Bisulfite kit (Qiagen). Pyrosequencing was then carried out using a PSQ 96MA system (Qiagen) with a Pyro Gold Reagent kit (Qiagen), and the results were analyzed using Pyro Q-CpG software (Qiagen). A cutoff value of 15% was used to define genes as methylation-positive. Using five classic CIMP markers (*MINT1*, *MINT2*, *MINT12*, *MINT31* and *MLH1*) and *CDKN2A* (*p16*), tumors were defined as CIMP-positive (three or more loci showed methylation) or CIMP-high (CIMP-H, four or more loci showed methylation). Methylation of *SMOC1*, *GALNT14*, *SFRP1*, *SFRP2*, *IGFBP7*, *SOX5* and long interspersed nucleotide element 1 (LINE-1) was also analyzed using bisulfite pyrosequencing. The primer sequences used were as previously reported [9,31,39].

### Immunohistochemistry

Immunohistochemical studies of β-catenin expression were performed as previously described with 39 serrated lesions, including 14 TSAs, 13 SSAs and 12 MVHPs [40]. A mouse anti-β-catenin monoclonal antibody (1:1000 dilution, Clone 14; BD Biosciences, San Jose, CA) was used. β-catenin expression was semi-quantitatively evaluated in tumor cells with β-catenin-positive nuclei, and positive nuclear accumulation was defined as staining of more than 10% of tumor cell nuclei throughout the lesions, as reported previously [40]. All slides were evaluated by two independent pathologists (YK and TM) who were blinded to the clinical and molecular data.

### Statistical analysis

Continuous data were analyzed using *t*-tests (for two groups) or ANOVA with a post hoc Tukey’s HSD test (for more than two groups). Fisher’s exact test and logistic regression were used to assess the association between categorical variables. Values of *P* < 0.05 were considered statistically significant. All statistical analyses were performed using SPSS 20 (IBM Corporation, Somers, NY) and GraphPad Prism 6 (GraphPad Software, La Jolla, CA).

## Results

### Clinicopathological characteristics of serrated lesions

The clinicopathological and molecular characteristics of the colorectal serrated lesions analyzed in this study are summarized in Tables 1 and 2. The majority of MVHPs (17/23, 74%) and SSAs (15/18, 83%) were located in the proximal colon, from the cecum to the transverse colon, while TSAs were more prevalent in the distal colon (27/37, 73%), especially the sigmoid colon and rectum (14 cases and 11 cases, respectively). On endoscopic observation, more than one-third (14/37, 38%) of TSAs were protruding, pedunculated (0-Ip) lesions and differed significantly from SSAs, most of which were protruding, sessile (0-Is) lesions (16/18, 88%) (Table 2).

**Table 2:** Clinicopathological and molecular characteristics of the respective serrated lesion.

	MVHP	SSA	TSA	*p* value (SSA vs TSA)
**No. of cases**	23	18	37	
**Sex (male/female)**	16/7	9/9	26/11	0.24
**Age (y, mean ± SD)**	66 ± 9.1	63.3 ± 10.6	65.8 ± 11.8	0.44
**Tumor location, n (bowel subsite)**				
Proximal (C/A/T)	17 (6/10/1)	15 (6/5/4)	10 (1/7/2)	<0.01
Distal (D/S/R)	6 (1/5/0)	3 (2/1/0)	27 (2/14/11)	
**Tumor size (mm, mean ± SD)**	11.3 ± 7.0	11.2 ± 4.7	12.2 ± 5.4	0.5
**Morphology, n (%)**				
0-Ip	0 (0)	1 (6)	14 (38)	0.01
0-Is	13 (57)	16 (88)	18 (49)	
0-IIa	10 (43)	1 (6)	5 (13)	
**Gene mutation/epigenetic alteration, n (%)**				
*BRAF* V600E mutation	14 (61)	14 (78)	24 (65)	0.37
*KRAS* mutation	4 (17)	1 (6)	11 (30)	0.08
*RNF43* mutation	3 (13)	3 (17)	14 (38)	0.13
*APC* mutation	1 (4)	0 (0)	7 (19)	0.08
WNT signaling associated genes	9 (39)	5 (28)	24 (65)	<0.01
CIMP	9 (39)	8 (44)	16 (43)	1
CIMP-high	5 (22)	4 (22)	10 (27)	1
*SMOC1* methylation	0 (0)	0 (0)	20 (54)	<0.01

MVHP, microvesicular hyperplastic polyp; SSA, sessile serrated adenoma; TSA, traditional serrated adenoma; C, cecum; A, ascending colon; T, transverse colon; D, descending colon; S, sigmoid colon; R, rectum; CIMP, CpG island methylator phenotype

### Targeted amplicon sequencing of colorectal serrated lesions

We performed semiconductor sequencing of all exons in 39 cancer-related genes that were previously detected in 78 colorectal serrated lesions and advanced CRCs. The sequencing overview, including reads, coverage, and uniformity of the read coverage distribution, is shown in S1 Table. Each FFPE sample underwent an average of 2.7 million sequencing reads after quality filtering. A mean coverage depth of 1722.5 reads (100.2–5126.0) per base was observed.

**Fig 1:**
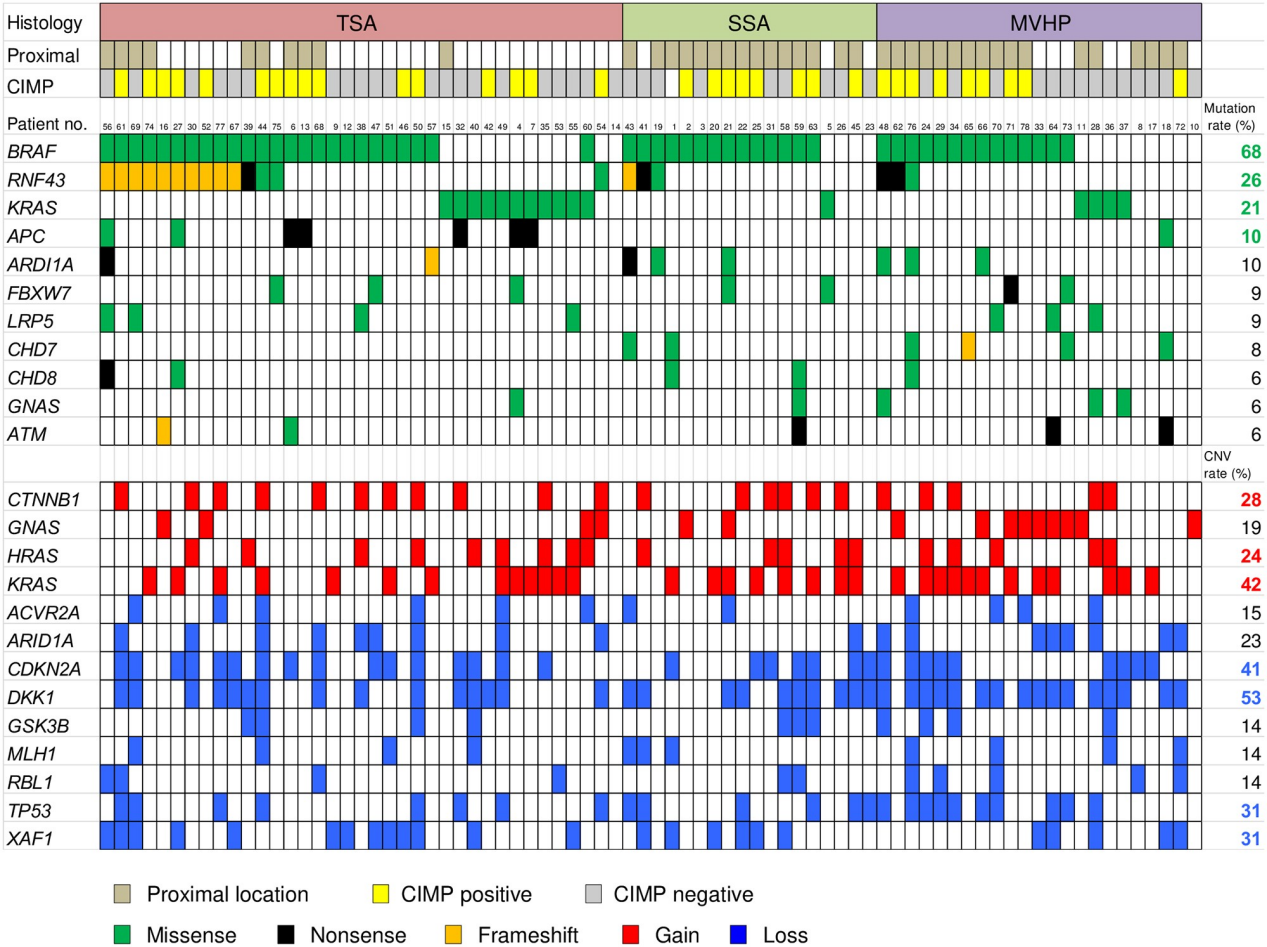
Summary of somatic mutations and CNVs across 78 serrated lesions. Profiles of gene mutations and CNVs within individual samples are grouped with respect to histological types. In the upper panel, the top row indicates the histology, the second row indicates the tumor location, and the third row indicates the CIMP status. Columns correspond to the individual cases. In the middle panels, frequently mutated genes, colored to indicate the type of mutation, and their mutational frequency are shown. In the lower panels, CNVs frequently detected in colorectal serrated lesions are shown.

**Fig 2:**
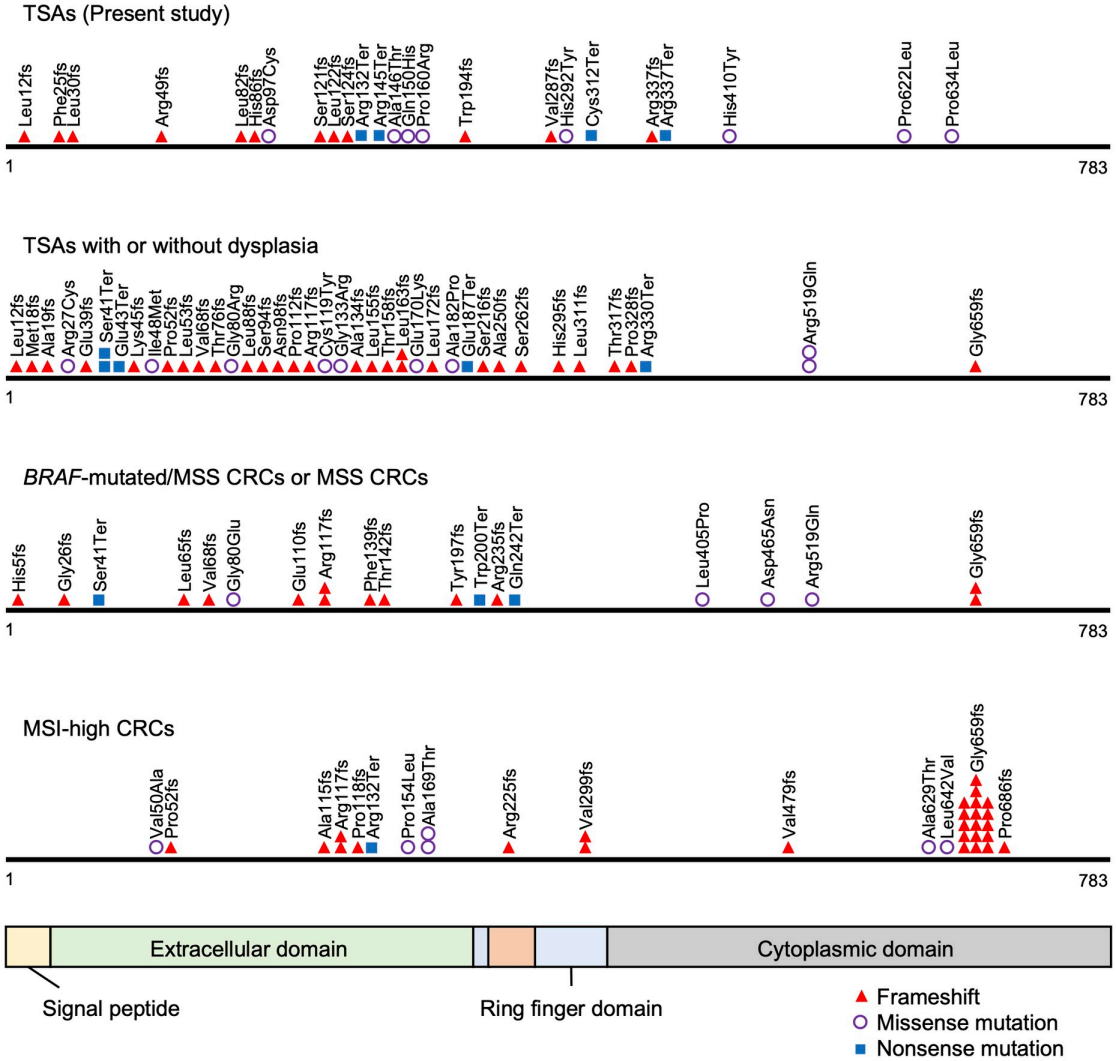
Distributions of *RNF43* mutations in detected in TSAs in the present study. Previously reported TSA mutations with or without dysplasia as well as MSS and MSI-high CRCs are shown for reference [23,24,41].

**Fig 3:**
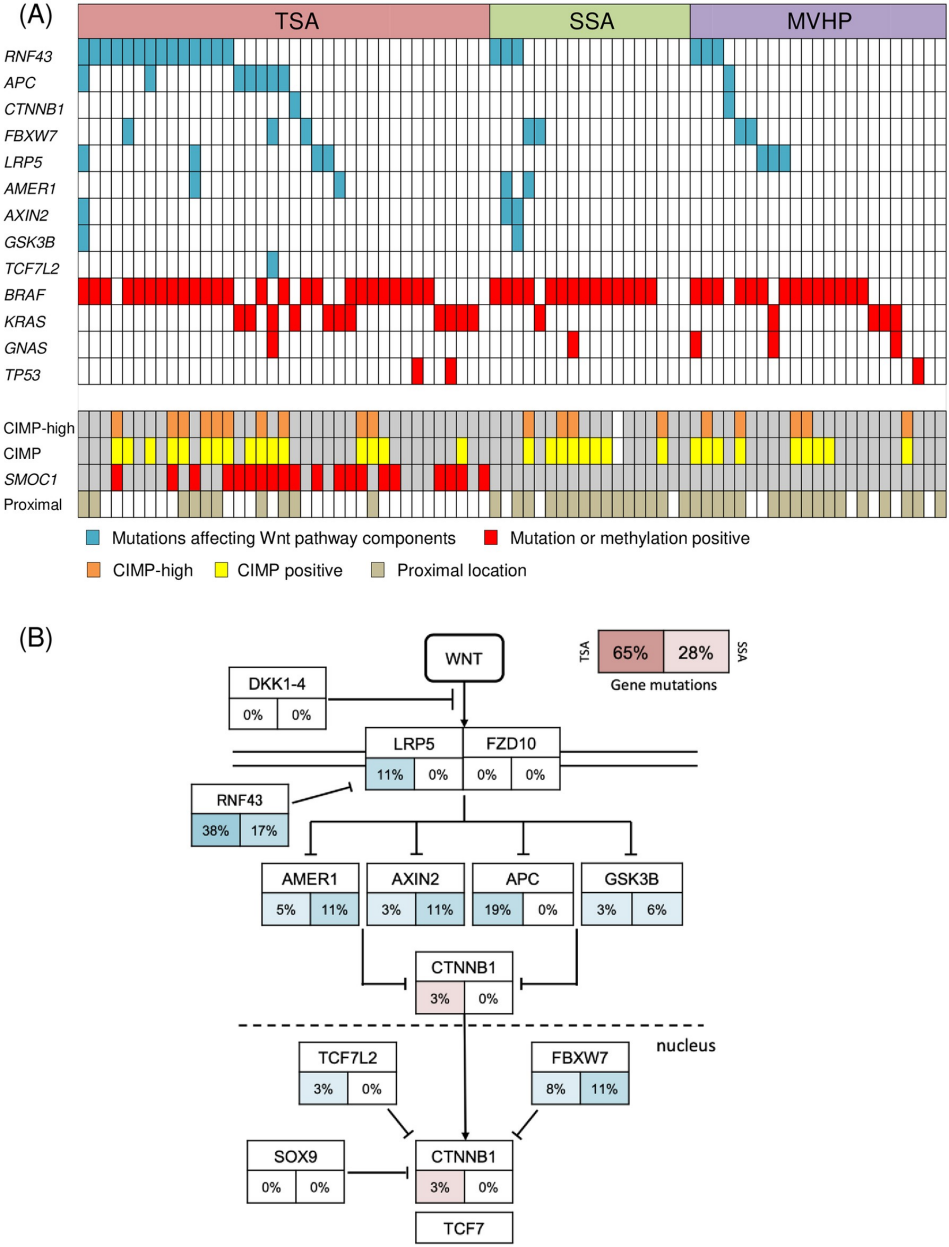
Gene mutations leading to dysregulation of Wnt signaling pathway in serrated lesions. (A) Representative mutation and methylation profile across 78 serrated lesions. Blue coloration indicates gene mutations affecting Wnt pathway components, while red coloration indicates positivity for gene mutations or methylation. (B) Frequencies of gene mutations leading to dysregulation of Wnt signaling in TSAs and SSAs. Alteration frequencies are expressed as percentages of all cases. Frequencies of gene mutations in TSAs are shown on the left, while those in SSAs are shown on the right. Red denotes activated genes and blue denotes inactivated genes.

All single nucleotide variants (SNVs) and insertions and deletions (InDels) detected through bioinformatics analysis underwent visual inspection using the IGV for confirmation. We identified a mean of 2.4 somatic nonsynonymous mutations (range 0–13) per sample (S2 Table). The 11 most commonly mutated genes in serrated lesions are depicted in Fig 1. *BRAF* was the most frequently mutated gene in serrated lesions (68%, 53 of 78 cases), followed by *RNF43* (26%), *KRAS* (21%), and *APC* (10%). *BRAF* V600E mutations were detected in 52 samples, and an N581S mutation was detected in 1 sample accompanied by a *KRAS* Q61H mutation (Patient 60 in Fig 1). The *BRAF* V600E mutation and *KRAS* mutation appeared to be mutually exclusive, as they were never detected in the same sample (Fig 1). *RNF43* mutations were detected in 38% of TSAs and 17% of SSAs. Nonsense or frameshift mutations in *RNF43* were seen in 30% of TSAs and 11% of SSAs. Most of the mutations were located upstream of the ring finger domain of *RNF43* (Fig 2). We also found *APC* mutations in 7 of 37 TSAs (19%), but none in SSAs. Protein-truncating (nonsense and frameshift) mutations of *APC* were detected in 14% of TSAs (Fig 1). Overall, the frequency of mutations in Wnt pathway components was significantly higher in TSAs than in SSAs (65% vs. 28%, P < 0.01) (Fig 3, Table 2). In addition, a *GNAS* R201H mutation was found in 1 TSA, while 1 SSA and 3 MVHPs harbored a *GNAS* V334G mutation, though the biological significance of the latter is unknown (Fig 3A, S2 Table).

### Target amplicon sequencing detects CNVs

We also detected CNVs in segments of the genome that could be duplicated or deleted from the sequencing data (Fig 1). In all samples, the genes most frequently affected by copy number gains were *KRAS* (42%), *CTNNB1* (28%), and *HRAS* (24%), while the genes most frequently affected by copy number losses were *DKK1* (53%), *CDKN2A* (41%), *TP53* (31%), and *XAF1* (31%). In addition, copy number loss at the *APC* gene locus was seen in one TSA (S2 Fig). In total, at least one CNV (loss or gain) affecting a Wnt pathway component was found in 49% of TSAs and 67% of SSAs. When considered together with the gene mutations, at least one genomic abnormality affecting genes associated with Wnt signaling was seen in 84% of TSAs and 78% of SSAs. Among these, the most frequently affected genes associated with Wnt signaling were *CTNNB1* (gain at 3p22.1), which encodes β-catenin, and *DKK1* (loss at 10q21.1), which encodes Dkk-1, a negative regulator of Wnt signaling (S2 Fig).

### Methylation analysis of CIMP markers, cancer-associated genes, and LINE-1

We next assessed methylation of CIMP markers and genes known to be frequently methylated in CRCs (S3 Table) [9]. When comparing TSA and SSA, the prevalence of CIMP statuses (CIMP-positive or CIMP-H) did not significantly differ (Table 2, Fig 3A). Notably, however, *SMOC1* methylation was detected in 20 of 37 TSAs (54%) but in none of the 18 SSAs tested (P < 0.01) (Table 2, Fig 3A). By contrast, the frequencies of lesions showing methylation of other cancer-associated genes did not significantly differ between TSAs and SSAs. There were no lesions in which *MLH1* methylation was positive in the present study.

We found that the levels of *SMOC1*, *SFRP1 and SFRP2* methylation were significantly higher in TSAs than in SSAs or MVHPs (S3 Fig). In addition, the level of *SOX5* methylation was significantly higher in TSAs than in MVHPs. Levels of LINE-1 methylation, which was measured to evaluate global DNA hypomethylation in the lesions, did not correlate with the histological types of serrated lesions (S3 Fig).

### Clinicopathological and molecular characteristics of TSAs with *KRAS* or *BRAF* mutation

We also compared clinicopathological and molecular characteristics of TSAs with *KRAS* or *BRAF* (V600E) mutation. It appears that TSAs with *BRAF* mutation were more likely to be located in the proximal colon than those with *KRAS* mutation, though the difference was not statistically significant (P = 0.12) (Table 3). Regarding gene mutations, *RNF43* mutation was frequently detected in TSAs with *BRAF* mutation, but not in TSAs with *KRAS* mutation (P < 0.01). Lesions with CIMP-H were also found only in TSAs with *BRAF* mutations (38% vs. 0%, P = 0.03). Although lesions positive for *SMOC1* methylation were seen in both groups, *SMOC1* methylation was significantly more prevalent among TSAs with *KRAS* mutations than those with *BRAF* mutations (82% vs. 38%, P = 0.03) (Table 3). In addition, levels of *SFRP1* methylation were significantly higher in TSAs with *KRAS* mutation than in those with *BRAF* mutation. On the other hand, levels of *IGFBP7* methylation was significantly higher in TSAs with *BRAF* mutation than those with *KRAS* mutation (S4 Fig).

**Table 3:** Clinicopathological and molecular characteristics of the *KRAS*- and *BRAF*- mutant TSAs.

	*BRAF* mutant TSA	*KRAS* mutant TSA	*p* value
**No. of cases**	24	11	
**Sex (male/female)**	16/8	9/2	0.45
**Age (y, mean ± SD)**	65.9 ± 8.7	63.1 ± 15.9	0.61
**Tumor location, n (bowel subsite)**			
Proximal (C/A/T)	9 (1/7/1)	1 (0/0/1)	0.12
Distal (D/S/R)	15 (2/9/4)	10 (0/4/6)	
**Tumor size (mm, mean ± SD)**	11.9 ± 4.5	12.1 ± 5.9	0.92
**Morphology, n (%)**			
0-Ip	10 (42)	3 (27)	0.26
0-Is	12 (50)	5 (46)	
0-IIa	2 (8)	3 (27)	
**Gene mutation/epigenetic alteration, n (%)**			
*RNF43* mutation	13 (54)	0 (0)	< 0.01
*APC* mutation	4 (17)	3 (27)	0.65
WNT signaling associated genes	17 (71)	6 (55)	0.35
CIMP	12 (50)	3 (27)	0.28
CIMP-high	9 (38)	0 (0)	0.03
*SMOC1* methylation	9 (38)	9 (82)	0.03

TSA, traditional serrated adenoma; C, cecum; A, ascending colon; T, transverse colon; D, descending colon; S, sigmoid colon; R, rectum; CIMP, CpG island methylator phenotype

### Immunohistochemistry

Of 39 serrated lesions analyzed, 12 (31%) showed positive nuclear accumulation, while 27 (69%) showed membranous expression of β-catenin. The abnormal nuclear accumulation was observed in 8/14 TSAs (57%), 1/13 (8%) of SSAs, and 3/12 (25%) MVHPs (S4 Table, S5 Fig). When compared with SSAs, the nuclear accumulation of β-catenin was more prevalent in TSAs (57% vs. 8%, P = 0.01). Across all lesions, the prevalence of nuclear β-catenin accumulation was significantly higher in lesions with *RNF43* mutations than in those without mutations (83% vs. 21%, P < 0.01). When we focused only on TSAs, the prevalence of nuclear β-catenin accumulation was significantly higher in TSAs with a *BRAF* V600E mutation than those with *KRAS* mutation (7/8 vs. 1/5, P = 0.01).

## Discussion

In the present study, we used targeted next-generation sequencing to assess the mutation of genes associated with Wnt signaling. We found that these mutations occurred with higher frequency in TSAs than SSAs, which is suggestive of the importance of Wnt signaling in the pathogenesis of TSAs. Sekine et al. reported genetic alterations that included *PTPRK-RSPO3* fusions among Wnt pathway components in 71% of TSAs [23]. If *PTPRK-RSPO3* fusions had also been investigated in the present study, the frequency of genetic alterations would have been even higher. *RNF43* encodes E3 ubiquitin ligase, which negatively regulates Wnt signaling. We detected *RNF43* mutations in 38% of TSAs and 17% of SSAs, which is consistent with earlier reports [23,24,26]. Most mutations, especially protein-truncating mutations, were situated upstream of the ring finger domain without clustering, as was shown in previous studies of TSAs with or without dysplasia [23,24]. Two major frameshift mutations (Arg117fs and Gly659fs) have been reported in CRCs with MSI [41], while CRCs with *BRAF* mutation/MSS had mutation profiles similar to TSAs [24,41]. This finding may indicate that TSAs belong to a carcinogenic pathway that is distinct from the SSA pathway (serrated-neoplasia pathway), making them possible precursors of *BRAF* mutated/MSS CRCs. We also sequenced the entire region of the *APC* gene and found protein-truncating mutations in 14% of TSAs, which is consistent with a report from Sekine et al. (13%) [23], though they only investigated frequently mutated regions of the gene. Protein truncating mutations of *RNF43* and *APC* were found to be mutually exclusive, suggesting the importance of both genes to Wnt signaling during carcinogenesis in TSAs. By contrast, the other genes encoding Wnt pathway components, including *CTNNB1*, *FBXW7*, *LRP5*, *AMER1*, and *AXIN2*, harbored mutations in only small fractions of tumors.

Previous studies have shown that there is wide variation in the rate of β-catenin positivity in both TSAs [21,23,27–29] and SSAs [29]. We showed in the present study that nuclear β-catenin accumulation is significantly more prevalent in TSAs than SSAs. This is likely associated with a high prevalence of mutations in the Wnt signaling pathway, especially in *RNF43*. These results may thus support the greater significance of *RNF43* mutation and Wnt signaling pathway activation in the carcinogenesis of TSAs than SSAs. Alternatively, it is possible that *RNF43* mutation represents subgroups in which Wnt signaling is activated by other genetic or epigenetic mechanisms.

Although an earlier study using a comparative genomic hybridization (CGH) microarray reported that CNVs were found only infrequently in colorectal precursor lesions including serrated lesions [9], CNVs in colorectal serrated lesions have not been thoroughly investigated. The present study demonstrated the frequent occurrence of CNVs at the *CTNNB1* and *DKK1* loci among Wnt signaling pathway associated genes. In addition, copy number losses at 17p (the *TP53* locus) are reportedly associated with progression of tumors from conventional-type adenoma to carcinoma and are frequently found in advanced CRCs [2,3]. This result may indicate the importance of *TP53* to carcinogenesis of serrated lesions. Because recent studies suggest that somatic CNVs at oncogenic loci are not always associated with gene expression [2,42,43], validation of the effect of CNVs through comparison with expression data is needed.

The prevalence of CIMP-positive or CIMP-H lesions did not significantly differ between SSAs and TSAs in this study. CIMP was detected in up to 79% of TSAs in previous studies [16,17]. One recent study reported that the prevalence of CIMP-H was significantly higher in SSAs than TSAs [44], which is inconsistent with our results. This difference likely reflects differences in the sample cohort, or may be due to a difference in the CIMP markers analyzed or the method used for methylation analysis (pyrosequencing vs. *MethyLight*). Interestingly, levels of DNA methylation in *SFRP1* and *SFRP2* were higher in TSAs than in SSAs or MVHPs. Epigenetic inactivation of SFRP family genes, including *SFRP1* and *SFRP2*, occurs early during CRC progression and enables constitutive Wnt signaling in CRCs [45]. It was recently reported that levels of *SFRP1* and *SFRP2* transcription correlate inversely with the methylation levels in samples of gastric mucosa, with or without *H*. *pylori* infection, as well as background mucosa in gastric cancers [46]. It is possible that *SFRP1* and *SFRP2* methylation contributes more significantly to carcinogenesis in TSAs than SSAs, although these phenomena may be influenced by the sample cohort, the degree of contamination by non-neoplastic cells, and methodology. We also analyzed the methylation status of *SMOC1* in a patient cohort different from the one examined in an earlier study [31]. We found that *SMOC1* methylation was highly specific for TSAs, and the methylation level was significantly higher in TSAs than SSAs. Thus, *SMOC1* methylation may be a potential marker to distinguish TSAs from other serrated polyps.

Recent reports suggest that TSAs belong to a heterogenous category and develop through at least two different neoplastic progression pathways. It is also suggested that lesions with *BRAF* mutation and those with *KRAS* mutation exhibit different clinicopathological and molecular characteristics [18–21]. In the present study, TSAs with *BRAF* mutation were preferentially located in the proximal colon, while those with *KRAS* mutation were preferentially located in the distal colon and rectum. In addition, TSAs with *BRAF* mutation were more likely to be CIMP-positive than those with *KRAS* mutation. CIMP-H positivity was only detected in TSAs with *BRAF* mutation, which is consistent with an earlier study [21]. Lesions with *RNF43* mutation were also found only in TSAs with *BRAF* mutation, while the prevalence of *SMOC1* methylation was significantly higher in TSAs with *KRAS* mutation than with *BRAF* mutation. The level of *IGFBP7* methylation is significantly higher in TSAs with *BRAF* mutation than with *KRAS* mutation, though the frequency of methylation-positive *IGFBP7* cases did not significantly differ between the two groups. *IGFBP7* has been shown to play a central role in *BRAF*-induced senescence and to be a direct target of TP53 [47]. Although methylation of *IGFBP7* has been investigated in specific histological types of serrated lesions [10,48], *IGFBP7* methylation status has not been compared between these two TSA subtypes. In addition to these differences in genetic and epigenetic alterations, the difference in the prevalence of β-catenin expression further supports there being two different neoplastic pathways for TSAs. Determining whether TSAs with *KRAS* or *BRAF* mutation belong to different neoplastic pathways with different malignant potentials will require further clinicopathological and molecular analyses.

The present study has several limitations, including a relatively small sample size, lack of data on fusion genes, and a lack of normal background samples for mutational analysis to rule out single nucleotide polymorphisms. Nonetheless, we were able to make several important observations. First, the mutational status of genes involved in Wnt signaling differs among colorectal serrated polyps, depending on TSA histology, which likely results in the differences in nuclear β-catenin expression. Second, we confirmed that *SMOC1* methylation is very specific to TSAs. Third, we detected significant differences in clinicopathological and molecular variables between TSAs with *KRAS* or *BRAF* mutation, which may indicate the presence of separate carcinogenic pathways among TSAs. By comparing gene expression data from CRCs and SSAs, an earlier study found that a particular subtype of CRCs with a poor-prognosis developed from serrated lesions [49]. Additionally, previous studies have also shown that there are similarities between the gene expression profiles of SSAs and those of MVHPs and MSI CRCs, which supports the concept of a serrated-neoplasia pathway [50,51]. Comprehensive gene expression studies together with analyses of the genetic and epigenetic alterations in TSAs and comparison of those data with other serrated polyps or CRCs could potentially establish the molecular carcinogenesis pathway in TSAs.

## Supporting information

S1 Fig List of genes in the custom AmpliSeq gene panel used in this study. (PDF)

S2 Fig Gene mutations and CNVs in association with Wnt signaling across 78 colorectal serrated lesions. (PDF)

S3 Fig Levels of methylation of the indicated genes and LINE-1 in the indicated histological types of colorectal serrated lesions. (PDF)

S4 Fig Levels of methylation of the indicated genes and LINE-1 in TSAs with *BRAF* or *KRAS* mutation. (PDF)

S5 Fig Representative views of hematoxylin and eosin (left) and β-catenin (right) expression in SSA and TSA. (A, B) SSA showing membranous localization of β-catenin. (C, D) TSA showing nuclear accumulation of β-catenin.
(TIF)

S1 Table Summary of targeted amplicon sequencing data. (XLSX)

S2 Table Somatic nonsynonymous mutations found in 78 colorectal serrated lesions. (XLSX)

S3 Table Methylation profiles and CIMP status in colorectal serrated lesions. (XLSX)

S4 Table Nuclear β-catenin expression in colorectal serrated lesions. (XLSX)

## Abbreviations

A: ascending colon
C: cecum
CGH: comparative genomic hybridization
CIMP: CpG island methylator phenotype
CIMP-H: CpG island methylator phenotype-high
CIN: chromosomal instability
CNV: copy number variation
CRC: colorectal cancer
D: descending colon
FFPE: formalin-fixed, paraffin-embedded
IGV: Integrative Genomics Viewer
InDel: insertion and deletion
LINE: long interspersed nucleotide elements
MSI: microsatellite instability
MSS: microsatellite stable
MVHP: microvesicular hyperplastic polyp
R: rectum
S: sigmoid colon
SNV: single nucleotide variant
SSA: sessile serrated adenoma
T: transverse colon
TSA: traditional serrated adenoma
