# Correction: Significance of gene mutations in the Wnt signaling pathway in traditional serrated adenomas of the colon and rectum

**DOI:** 10.1371/journal.pone.0231404

**Published:** 2020-04-01

**Authors:** The PLOS ONE Staff

A grant number from the funder JSPS KAKENHI is missing from the Funding statement. The missing grant number is: 16H06279 (PAGS) (TT). The publisher apologizes for the error.
