# Supplementary material for: Significance of gene mutations in the Wnt signaling pathway in traditional serrated adenomas of the colon and rectum
Source: PLoS One. 2020 Feb 24;15(2):e0229262. doi: 10.1371/journal.pone.0229262 (PMC7039454; doi:10.1371/journal.pone.0229262)
Supplement: S1 Fig — (PDF) [file pone.0229262.s001.pdf]

S1 Figure. List of genes in the custom Ampliseq gene panel used in this study.

ACVR2A	*DKK2*	*NRAS*
AMER1	*DKK3*	*PIF1*
APC	*DKK4*	*PIK3CA*
ARID1A	*FBXW7*	*PTEN*
ATM	*FZD3*	*RBL1*
AXIN2	*FZD10*	*RNF43*
BMP4	*GNAS*	*SMAD4*
BRAF	*GSK3B*	*SOX9*
CDKN2A	*HRAS*	*TCF7L2*
CHD7	*KRAS*	*TELO2*
CHD8	*LRP5*	*TGFBR2*
CTNNB1	*MLH1*	*TP53*
DKK1	*MSH6*	*XAF1*
