# Supplementary material for: Significance of gene mutations in the Wnt signaling pathway in traditional serrated adenomas of the colon and rectum
Source: PLoS One. 2020 Feb 24;15(2):e0229262. doi: 10.1371/journal.pone.0229262 (PMC7039454; doi:10.1371/journal.pone.0229262)
Supplement: S3 Fig — (PDF) [file pone.0229262.s003.pdf]

S3 Figure. Levels of methylation of the indicated genes and LINE-1 in the indicated histological types of colorectal serrated lesions.

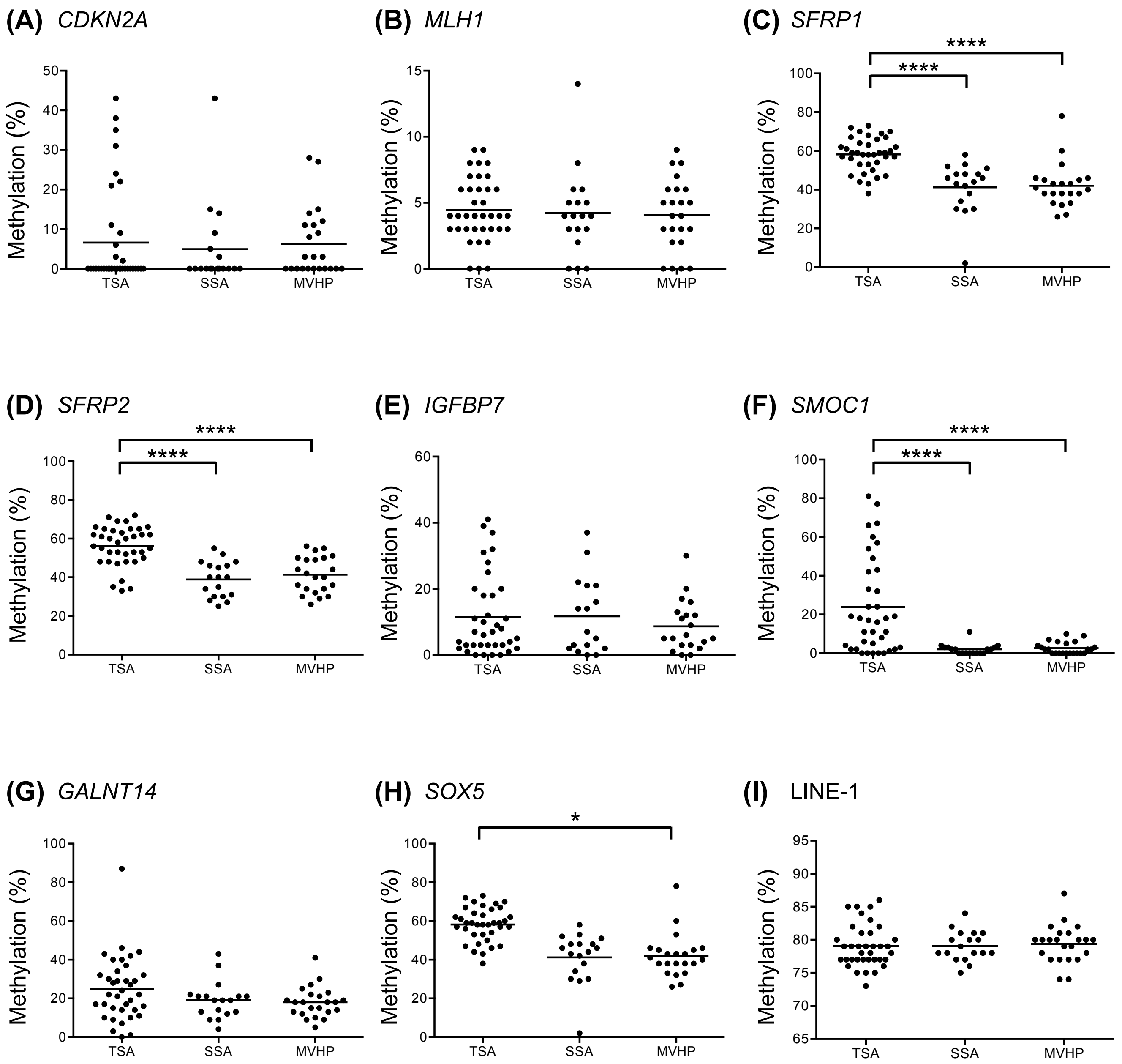

*$P < 0.05$, ****$P < 0.001$.
