# Supplementary material for: Significance of gene mutations in the Wnt signaling pathway in traditional serrated adenomas of the colon and rectum
Source: PLoS One. 2020 Feb 24;15(2):e0229262. doi: 10.1371/journal.pone.0229262 (PMC7039454; doi:10.1371/journal.pone.0229262)
Supplement: S4 Fig — (PDF) [file pone.0229262.s004.pdf]

S4 Figure. Levels of methylation of the indicated genes and LINE-1 in TSAs with *BRAF* or *KRAS* mutation.

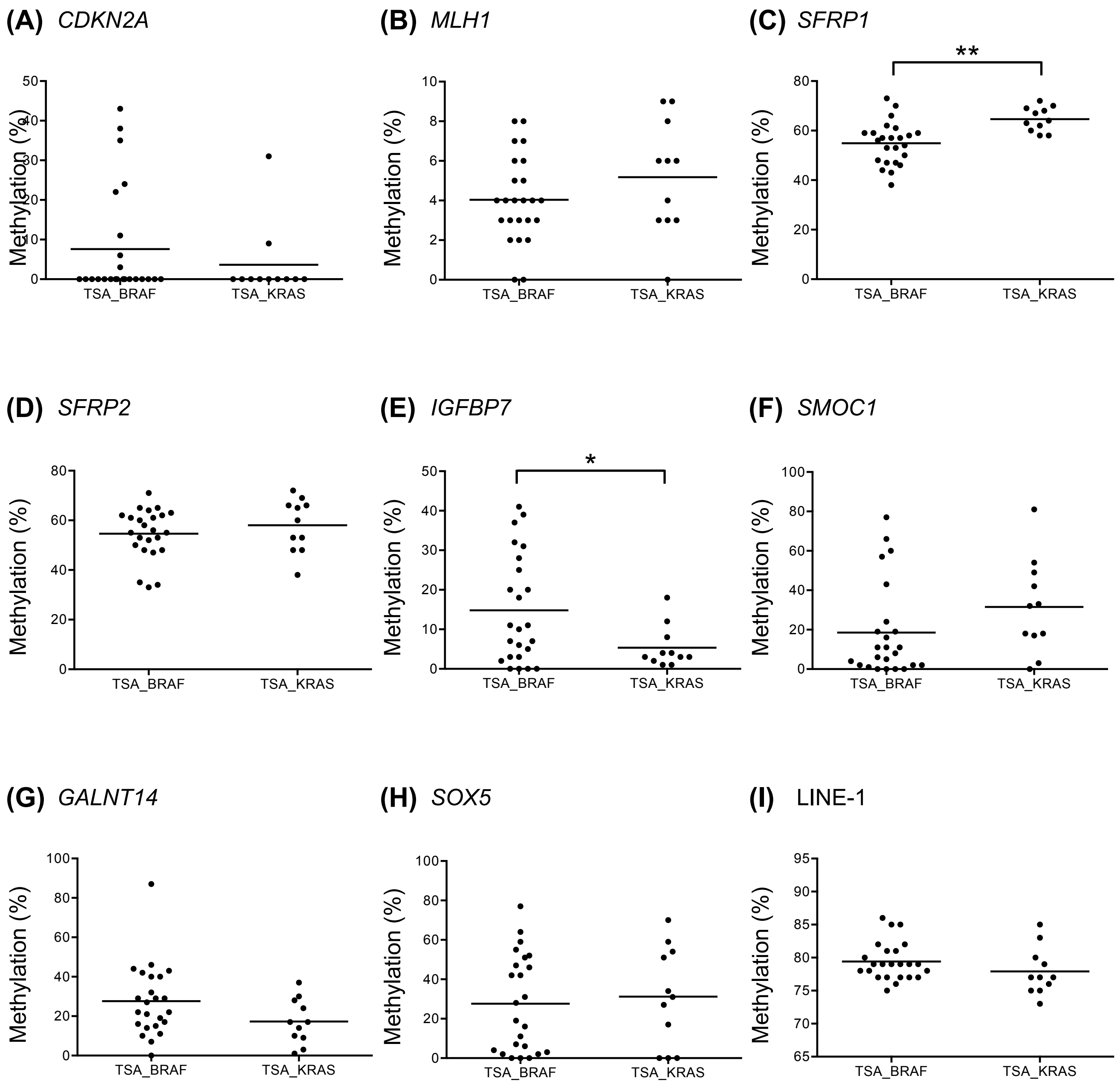

*P < 0.05, **P < 0.01.
