# Supplementary figures and images for: Significance of gene mutations in the Wnt signaling pathway in traditional serrated adenomas of the colon and rectum
Source: PLoS One. 2020 Feb 24;15(2):e0229262. doi: 10.1371/journal.pone.0229262 (PMC7039454; doi:10.1371/journal.pone.0229262)

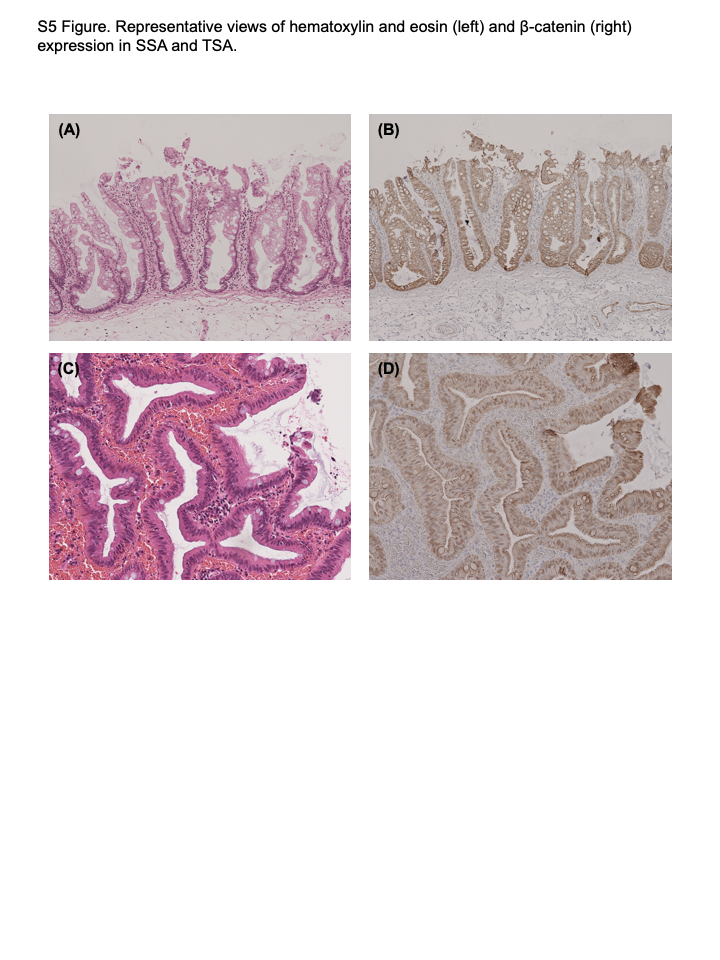

Supplement: S5 Fig — (A, B) SSA showing membranous localization of β-catenin. (C, D) TSA showing nuclear accumulation of β-catenin. (TIF) [file pone.0229262.s005.tif]
