# Supplementary material for: Significance of gene mutations in the Wnt signaling pathway in traditional serrated adenomas of the colon and rectum
Source: PLoS One. 2020 Feb 24;15(2):e0229262. doi: 10.1371/journal.pone.0229262 (PMC7039454; doi:10.1371/journal.pone.0229262)

S2 Figure. Gene mutations and CNVs in association with Wnt signaling across 78 colorectal serrated lesions.

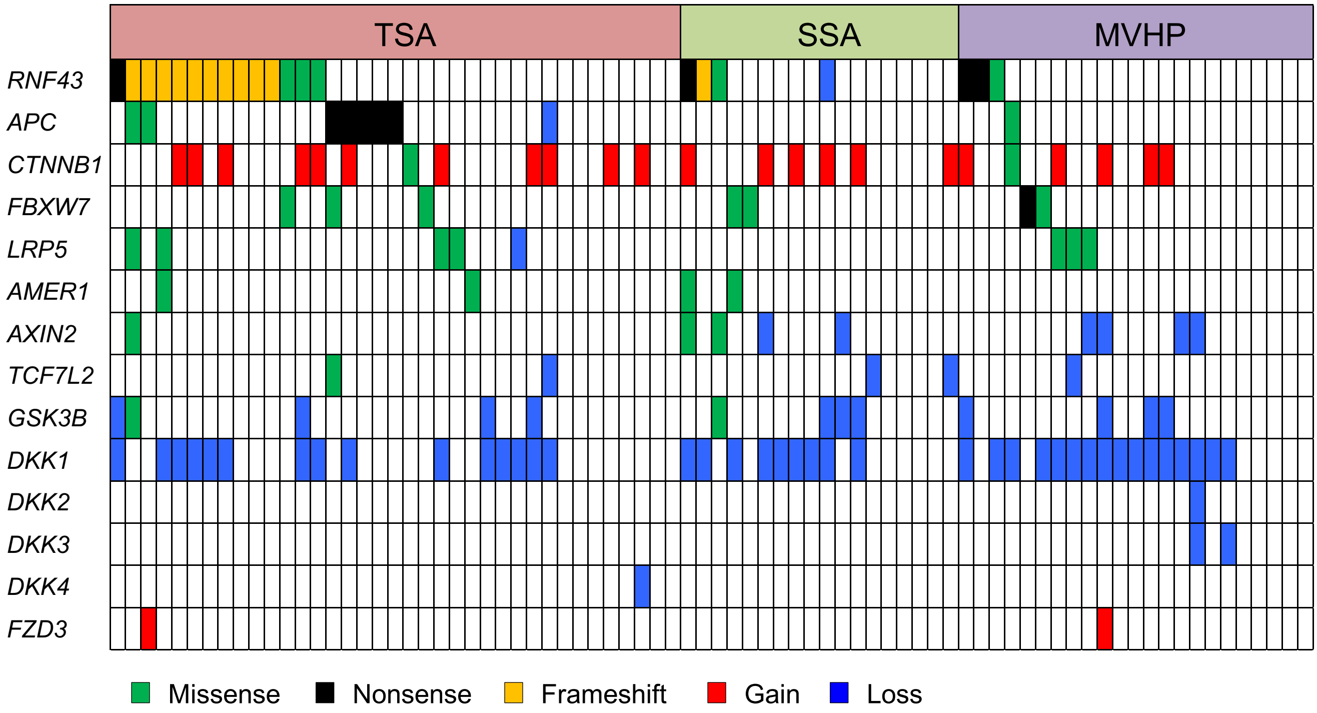

Supplement: S2 Fig — (PDF) [file pone.0229262.s002.pdf]
